# Convergence in public health expenditure across the Sub-Saharan African countries: does club convergence matter?

**DOI:** 10.1186/s13561-021-00316-0

**Published:** 2021-06-15

**Authors:** Ousmane Traoré

**Affiliations:** Department of Economics (UFR/SEG), Université Thomas Sankara (UTS), 12 P.O. BOX 417, Ouagadougou 12, Burkina Faso

**Keywords:** Health expenditure, Club convergence, log*t* regression, Sub-Saharan Africa

## Abstract

**Background:**

Sub-Saharan African (SSA) countries that currently face enormous healthcare challenges have implemented national health policies focusing on regional or international health commitments. These health commitments generally promote new healthcare financing policies (e.g., health insurance, user fee exemption and results-based financing) with the objective of providing ever-larger population cohorts with human capital and better health in particular. To achieve this, governments must involve themselves more fully in their respective healthcare sectors through the mobilisation of public funding.

**Objective:**

This paper aims to examine convergence in health expenditure throughout SSA. The findings of club convergence will allow a robust comparison of health indicators between countries and will be suitable for the adjustment of health policies to foster the efficiency of such policies at the regional and/or country level. Such findings could also help with the conception and implementation of health policies at the regional level.

**Methods:**

We used the methodology of convergence analysis based on dynamic factor modelling leading to the log*t* regression to test for full convergence, club convergence and club clustering of health expenditure on a balanced panel of 44 countries in Sub-Saharan Africa spanning the period from 2000 to 2016.

**Results:**

Overall, our results do not support the hypothesis that all SSA countries converge to a single equilibrium state regarding public health expenditure. When testing for club convergence, the results highlight eight convergence clubs and one group of diverging countries. Indeed, performing the club clustering algorithm reveals the existence of three convergence clubs and the diverging group. The three clubs consist of 12, 14 and 14 members, respectively, where convergence is found to occur among different regional economic organisations.

**Conclusion:**

Our findings indicate that SSA governments should increase spending on healthcare in order to align their healthcare systems with a global convergence model. To foster the convergence to a single equilibrium state in public health expenditure, attention could be paid to strengthening integration within the various regional economic organisations and to the coordination and integration of healthcare policies within and across convergence clubs throughout SSA.

**Supplementary Information:**

The online version contains supplementary material available at 10.1186/s13561-021-00316-0.

## Introduction

### Background

Healthcare is an increasingly important sector of the global economy, as reflected in the ever-increasing levels of health expenditure around the world [1]. Consequently, health expenditure has become a major concern in several studies in the field of health economics. Research in this field can be classified into two main groups. The first research group analyses the determinants of health expenditure [2–4] or the nature of health (luxury or necessity) by analysing the long-term cointegration relationship of per capita health expenditure and gross domestic product (GDP) [5–11]. The second research group recently highlights a growing interest in convergence analysis of health expenditure by investigating the convergence hypothesis of health expenditure among a panel of countries or among separate regions within a country [1, 12–17]. Borrowing the concept of economic convergence from neoclassical growth theory, health economists in this second research group have considered convergence of health care expenditure between the OECD countries or between the EU member countries [13].

Health expenditure convergence has several policy implications. The presence of convergence in public expenditure could be used as an indicator of the rational distribution of outcomes emerging from the implementation of several economic policies, either across countries or across the regions of a particular country [16]. Hitiris [4] stated that because health expenditure depends primarily on the level of economic development and the structure of a population, only a convergence in economic performance and living standards can ultimately lead to a convergence of health expenditure standards. Wang [13] argued that health expenditure is an increasingly important component of consumption because this type of expenditure will show signs of convergence across regions if income also converges. Additionally, given that cross-regional policy coordination often accompanies resource redistribution, this policy may also drive the convergence of health expenditure in the absence of income convergence. The main contributing factors that underlie convergence in health expenditure are the integration of healthcare markets, and common policies that promote health, good working conditions, coordination of medical research, insurance coverage and the diffusion of healthcare technologies and products [1].

### Motivation and project aims

Atiim et al. [18] highlighted four approaches which influence how much governments spend on health and thereby, could underlie research on convergence in health expenditure between countries. The peer pressure approach recommends the amount of health expenditure for a government according to other governments with similar characteristics such as income or epidemiological profile. The second approach concerns the political economy approach for which health spending may be influenced by the interplay of political and economic forces which determine budget priorities. The third approach is the production function approach which estimates changes in health spending based on health status after controlling socioeconomic and demographics factors. In line with this approach, Atiim et al. [18] proposed framework that seeks to account for both macro-level and local production of health. Fourth, the budget approach generates levels of health spending through identifying health service demand and the price of health services. In the particular context of SSA countries, several health policies are motivated by the aforementioned four approaches and could give way to researches on the convergence of health expenditure.

A large number of healthcare systems in SSA countries are currently grossly underfunded. A critical threshold of sustained investment in healthcare systems must be met by every country [19]. To this end, integrated healthcare markets and common policies promoting health are being implemented throughout SSA, meaning that African governments are becoming increasingly involved in diverting resources to their respective healthcare systems, thereby fulfilling the pledges made in the Abuja Declaration of committing 15% of their budgets to healthcare [19].

Additionally, most SSA countries continue to experience rapid population increases, which will pose severe challenges for the socioeconomic developmental prospects of the region, requiring countries to provide ever-larger numbers of young people with the human capital (e.g., health, nutrition, education) that they require [20]. However, 31 African countries had a total annual per capita health expenditure of $20 or less in 2001 [21]. In this context, investigating the pattern of public health expenditure per capita in SSA since 2001 will be both an important evaluative measure and a contribution to SSA countries’ commitment to improving the health of their respective populations.

Since its inclusion as one of the health-related Sustainable Development Goals (SDGs) set by world leaders in September 2015, Universal Health Coverage (UHC), in line with insurance coverage, has cemented its place in the SSA health policy agenda [21]. By implementing the UHC the SSA countries are involved in the common challenge of providing health care to their populations who generally face a similar epidemiological profile [22]. One could assume, however, that achieving health expenditure convergence earlier than 2015 could have strengthened the implementation of UHC in SSA countries and could have enabled a regional dimension of UHC in SSA. This is because convergence in public health expenditure can facilitate (or may be a prior condition of) the coordination of health coverage systems mostly between neighbouring countries or within integrated SSA economies.

Indeed, regional integration is being seen as a rational response to the difficulties faced by a continent with many small national markets and landlocked countries. As a result, African governments have embraced regional integration as an important component of their development strategies by creating a large number of regional integration arrangements, several of which have significant overlapping membership [23]. After accounting for these mergers, there are four main SSA integration groups: the Common Market for Eastern and Southern Africa (COMESA), the Economic Community of Central African States (ECCAS), the Economic Community of West African States (ECOWAS) and the Southern African Development Community (SADC). In this context, it needs to be determined whether the SSA countries’ commitment to the funding and improvement of healthcare tend towards single or multiple equilibrium states.

Overall, there is a need for research on health expenditure convergence in the SSA region, insofar as most studies on health expenditure convergence have focused on developed economies, with a current scarcity of such research on sub-Saharan economies. To our knowledge, only Odhiambo et al. [24] have investigated the convergence of health expenditure in SSA using a dynamic panel model. Therefore, this paper aims to examine the convergence in per capita government health expenditure in SSA countries. From this perspective, the paper aims to test the hypothesis of whether there is a global convergence or club convergence of public health expenditure in SSA.

### Background to the convergence methods

Convergence analysis in economics has largely been confined to macro-indicators such as GDP income, and has been applied within identifiable regions around the world or between developed and developing countries [25]. Therefore, the concept of convergence is relevant in growth literature and generally highlights three different types of convergence [26]. Baumol [27] introduced the concept of *β*-convergence, which empirically implies a negative relationship between the growth rate of the variable of interest and its initial level. Thus, convergence means that countries with a high initial level of a given outcome have a lower growth rate than countries with a lower initial level. However, many researchers have criticised *β*-convergence, such as De Long [28] and Quah [29] who show that this approach can lead to spurious levels of convergence. In other words, convergence can be proven by testing, even when it does not exist.

In contrast, Barro and Sala-i-Martin [30] developed the *σ*-convergence concept, which is measured by the variation in the sample standard deviation. The convergence of a log-transformed outcome can be observed when its cross-sectional deviation decreases over time. Overall, *β*-convergence remains a necessary but insufficient condition for *σ*-convergence [31].

The third type of convergence is stochastic convergence. Here, the analysis is focused on the persistence of shocks on the outcome of interest. The concept of stochastic convergence is a time-series notion of convergence [29, 30]. Stochastic convergence suggests that the shocks in the logarithm of a per capita outcome relative to its average of the sample are temporary.

Previous studies on convergence of health expenditure focused on the above concepts of convergence. Narayan [12] examined the ‘catch-up’ hypothesis in health expenditure growth, i.e. whether the per capita health expenditures of the UK, Canada, Japan, Switzerland and Spain converged with the per capita health expenditure of the USA from 1960 to 2000. Narayan [12] used unit root testing, namely the Lagrange multiplier (LM) univariate and panel approaches that allowed for, at most, two structural breaks. This method failed to find evidence of convergence when structural breaks were not incorporated. Payne et al. [1] examined the stochastic conditional convergence of per capita health expenditure among 19 Organisations for Economic Co-operation and Development (OECD) countries between 1972 and 2008. The methods that the authors used were unit root tests, which allowed for two endogenously determined structural breaks. Their results showed health expenditure convergence among most OECD countries. Wang [13] analysed the convergence of health expenditure in the USA using *β*-convergence and *σ*-convergence as well as convergence based on time-series evidence. The results, relative to the time-series evidence of convergence in health expenditure and its components by cluster, showed the existence of asymptotically perfect and asymptotically relative convergence clubs. In line with the time-series evidence of convergence, Lee and Tieslau [32] applied the panel LM unit root tests using the level and trend shifts method and found evidence of stochastic convergence in health expenditure among OECD countries. In the context of Chinese counties, Zhang et al. [17] showed that health expenditures converge following *σ*-convergence and following both long-term and short-term *β*-convergence. They concluded that these findings indicated the effectiveness of the capitation subsidy method in the New Rural Cooperative Medical Scheme on narrowing disparities in government health expenditure between counties.

Regarding stochastic convergence, Phillips and Sul [33] developed a new approach: the log*t* regression test, which allows the testing of the convergence hypothesis based on a non-linear time-varying factor model. Indeed, the traditional convergence tests for economic growth have some pitfalls when heterogeneous transitions exist [34]. Using the log *t* test, Panopoulou and Panteledis [14] analysed the convergence of health expenditure among 19 OECD countries between 1972 and 2006 and found evidence of convergence in per capita health expenditure for 17 of the countries, while the USA and (to a lesser degree) Norway followed a different path. The results also indicated that convergence in health expenditure among the 17 OECD countries did not lead to a convergence in health outcomes. Du [35] highlighted some performance issues regarding the new approach proposed by Phillips and Sul [33], which considers heterogeneous agent behaviour and its evolution. Additionally, they found that the test does not make any particular assumptions concerning trend stationarity or stochastic non-stationarity, thereby being robust to the stationarity property of the series.

A common but crucial concern regarding convergence analysis is the possible existence of convergence clubs [35]. In this issue, traditional studies typically divided all countries into subgroups based on prior information (e.g., geographical location or institution) and then tested the respective convergence hypothesis for each subgroup. Another important merit of Phillips and Sul’s [33] approach of convergence, contrary to the traditional approach mentioned above, is related to its inherent new algorithm based on the relative transition parameter by identifying clusters of convergence subgroups.

In summary, the background on the methodological approaches of convergence highlights the merits of the log *t* regression test for a robust analysis of convergence. Our paper uses this method to analyse convergence in health expenditure per capita in the SSA. The rest of the paper is organised as follows. Section 2 presents the methods, Section 3 describes the data, Section 4 presents the results while Section 5 discusses them and Section 6 concludes the paper.

## Methods

### Convergence conceptual framework: dynamic factor modelling

In this paper, we use the econometric convergence analysis and club clustering proposed by Phillips and Sul [33] to analyse convergence in health expenditure in SSA. Following Phillips and Sul’s [33] conceptual framework, panel data, which included log national income data, regional log income data, regional consumer price index data and personal survey income data (among other factors), are often initially and usefully decomposed into two main components: a permanent common component and a transitory component. The permanent common component is assumed to give rise to cross-section dependence. Additionally, it is usefully assumed that the above two components of panel data may contain a mixture of both common and idiosyncratic components. The dynamic factor modelling proceeds with an initial decomposition of the panel data as follows:

1 $$ {X}_{it}={g}_{it}+{a}_{it};\kern0.5em i=1,\dots, N\kern1em \mathrm{and}\kern1em t=1,\dots, T, $$

where *X*_*it*_ is the natural logarithm of per capita health expenditure of panel unit *i* at time *t*, *g*_*it*_ represents the systematic components (i.e. the permanent common components of health expenditure in the whole panel) and *a*_*it*_ is the transitory component.

Because the systematic components and the transitory components may contain both common and idiosyncratic dimensions, common components can be separated from idiosyncratic components in the panel.

The second decomposition made in the dynamic factor modelling leads to the time-varying factor representation as follows:

2 $$ {X}_{it}=\left(\frac{g_{it}+{a}_{it}}{\mu_t}\right){\mu}_t={\delta}_{it}{\mu}_t\kern0.75em \mathrm{for}\ \mathrm{all}\ i,t. $$

According to this transformation, health expenditure *X*_*it*_ is decomposed into two time-varying components: a single common component *μ*_*t*_ and a time-varying idiosyncratic element *δ*_*it*_, which is a measure of the distance between the country outcome *X*_*it*_ and the common component. The evidence in SSA suggests that, infectious diseases, childhood illnesses, and maternal related deaths were responsible for close to 70% of the burden of disease in most countries in the region [22]. Other, population in SSA are dealing with the rise of noncommunicable diseases (NCDs). Diabetes and depression are so estimated to have risen by 88 and 61% respectively between 1990 and 2010 in SSA and the increasing burden of NCDs in SSA calls for targeted public health interventions [18]. Thus, the above common health risk factors in SSA may generate the common component of health expenditure. Furthermore, emerging NCDs disproportionately affect vulnerable groups such as women, and the poor living in urban slums [36]. Such disparities in health within and across countries may underlie the idiosyncratic element of health expenditure.

If *μ*_*t*_ represents a common trend component in log health expenditure per capita, then *δ*_*it*_ measures the relative share of country *i* at time *t* in health expenditure as a common trend. In other words, the coefficient *δ*_*it*_ measures the transition path of a country to the common steady-state health expenditure path determined by *μ*_*t*_ [34]. The representation (2) is a time-varying factor model in which *μ*_*t*_ is assumed to have some deterministic or stochastically trending behaviour that dominates the transitory component *a*_*it*_ over the long term. Indeed the specification allows for the possibility that some countries may diverge from the common health expenditure path, i.e. the common component *μ*_*t*_ of health expenditure, while others may converge towards it.

To meet the diverging or converging properties, the decomposition must allow testing for convergence by testing whether the idiosyncratic component converges to a constant, *δ*. To attempt this objective, we can use ratios instead of differences as a way of eliminating the common component. Phillips and Sul [34] propose an alternative approach to modelling the transition path by defining a relative transition coefficient, *h*_*it*_, which is easily derived from representation (2) as follows:

3 $$ {h}_{it}=\frac{X_{it}}{\frac{1}{N}\sum \limits_{i=1}^N{X}_{it}}=\frac{\delta_{it}}{\frac{1}{N}\sum \limits_{i=1}^N{\delta}_{it}}. $$

The relative transition parameter *h*_*it*_, like *δ*_*it*_, traces out the transition path for health expenditure of country *i*, but relative to the panel average. Equation (3) indicates that the cross-sectional mean of *h*_*it*_ is unity, and the cross-sectional variance of *h*_*it*_ satisfies the following condition:

$$ {H}_t=\frac{1}{N}\sum \limits_i^N{\left({h}_{it}-1\right)}^2\to 0\kern1em \mathrm{if}\kern1em \lim {\delta}_{it}=\delta \kern1em \mathrm{for}\ \mathrm{all}\kern0.5em i. $$

### The Convergence test

The convergence in health expenditure requires the following condition:

$$ \lim \frac{X_{it}}{X_{jt}}=1\kern1.25em \mathrm{for}\ \mathrm{all}\kern0.5em i\kern0.5em \mathrm{and}\kern0.5em j. $$

This condition is the relative convergence, which is equivalent to the convergence of the time-varying, factor-loading coefficient [33]. The loading coefficient *δ*_*it*_ is then decomposed as follows:

$$ {\delta}_{it}={\delta}_i+{\sigma}_{it}{\xi}_{it}\kern1em \mathrm{where}\kern1em {\sigma}_{it}=\frac{\sigma_i}{L(t){t}^{\alpha }},t\ge 1,{\sigma}_i>0\kern1.25em \mathrm{for}\ \mathrm{all}\kern0.5em i. $$

*L*(*t*) is a slowly varying function and can take the forms log*t*, log^2^(*t*) or log{log(*t*)}. By applying a Monte Carlo simulation, Phillips and Sul [33] show that the form log(*t*) leads to the least amount of size distortion and the best test power.

The test for the hypothesis of convergence is built on the following log(*t*) regression model:

4 $$ \log \left(\frac{H_1}{H_t}\right)-2\log \left\{\log (t)\right\}=a+b\log (t)+{\varepsilon}_t\kern0.62em \mathrm{for}\kern0.62em t=\left[ rT\right],\left[ rT\right]+1,\dots, T;\kern0.5em r\in \left[0.2,0.3\right] $$

With *b* = 2*α*, and for a small or moderate sample (*T* ≤ 50), *r* is set to 0.3.

Thus, the hypotheses are set as the following:

$$ {\displaystyle \begin{array}{c}{H}_0:{\delta}_i=\delta \leftrightarrow \alpha >0\\ {}{H}_A:{\delta}_i\ne \delta \leftrightarrow \alpha <0\end{array}}. $$

The tests imply a one-sided test, with the limit distribution of the regression *t* statistic being:

$$ {t}_b=\frac{\overset{\frown }{b}-b}{s_b}\Rightarrow N\left(0,1\right) $$

The null hypothesis of convergence is rejected if *t*_*b*_ <  − 1.65.

### The convergence club clustering and club merging

Rejection of the null hypothesis of convergence does not imply that there is no evidence of convergence in any of the panel subgroups. Convergence clusters may exist around separate points of equilibria or steady-state growth paths, as well as both convergence clusters and divergent members being present in the full data panel [33].

Phillips and Sul [33] provide an empirical algorithm that identifies subgroups of different states that converge to different equilibria. Schnurbus, Haupt, and Meir [37] showed that Phillips and Sul’s [33] algorithm could produce a non-conservative conclusion of the convergence hypothesis when it is stopped too early. Therefore, the authors propose simple adjustments to the algorithm which also allow its applicability to other data. Du [35] provides a more detailed description of the adjusted algorithm as follows.

- **Cross-section sorting (Step 1)**. In the first step, individuals (i.e. the countries in this paper) in the panel are sorted in decreasing order according to their observations over the last period. Individuals are indexed with their orders {1, …, *N*}. The ordering is based on the final observation of each series because evidence of convergence will, in general, be most apparent in recent years [26].
- **Core group formation (Step 2)**. Step 2 consists of two sub-steps. (2.1) Find the first *k* such that the test statistic of the log*t* regression *t*_*k*_ >  − 1.65 for the subgroup with individuals {*k*, *k* + 1}. If there is no *k* satisfying *t*_*k*_ >  − 1.65, we can stop the algorithm and conclude that there are no convergence subgroups in the panel. (2.2) Start with the *k* identified in sub-step (2.1) and perform the log*t* regression for the subgroups with individuals {*k*, *k* + 1, …, *k* + *j*} with *j* ∈ {1, …, *N* − *k*}. Choose *j*^∗^ such that the subgroup with individuals {*k*, *k* + 1, …, *k* + *j*^∗^} yields the highest value of the test statistic. Individuals {*k*, *k* + 1, …, *k* + *j*^∗^} form a core group.
- **Sieve individuals for club membership (Step 3)**. In the first sub-step of Step 3 (3.1), we can form a complementary group ${G}_{j^{\ast}}^C$ which contains all the remaining individuals not included in the core group of Step 2. Add one individual from ${G}_{j^{\ast}}^C$ at each time to the core group and run the log*t* test. Include the individual in the club candidate group if the test statistic is greater than the critical value *c*^∗^.
  Phillips and Sul [33] recommend that *c*^∗^ be set equal to zero for sample sizes of *<* 50 in order to ensure that the test is highly conservative. (3.2) Run the log*t* test for the club candidate group identified in sub-step (3.1). If the test statistic ${\hat{t}}_b$ is greater than −1.65, the initial convergence club is obtained. If not, Phillips and Sul [33] advocate raising the critical value *c*^∗^ and repeating sub-steps (3.1) and (3.2) until ${\hat{t}}_b>-1.65$. Schnurbus, Haupt, and Meier [37] propose adjusting this step as follows. If the convergence hypothesis does not hold for the club candidate group, sort the club candidates with respect to decreasing ${\hat{t}}_b$ obtained in sub-step (3.1). If there are some ${\hat{t}}_b>-1.65$, add the individuals with the highest value of ${\hat{t}}_b$ to form an extended core group. Add one individual from the remaining candidates at a time, run the log*t* test, and denote the test statistic ${\hat{t}}_b$. If the highest value of ${\hat{t}}_b$ is not greater than −1.65, stop the procedure; the extended core group will then form an initial convergence club. Otherwise, repeat the above procedure to add the individual with the highest ${\hat{t}}_b$.
- **Recursion and stopping rule (Step 4)**. Form a subgroup of the remaining individuals that were not sieved by Step 3 and perform the log*t* test for this subgroup. If the test statistic is greater than −1.65, the subgroup forms another convergence club. Otherwise, repeat steps 1–3 on this subgroup.
- **Club merging (Step 5)**. Perform the log*t* test for all pairs of the subsequent initial clubs. Merge those clubs fulfilling the convergence hypothesis jointly. Schnurbus, Haupt, and Meier [37] developed the iteration of club merging as follows. For *I* initial clubs, {1, 2, …, *I*}, run the log*t* test for initial clubs 1 and 2. If they fulfil the convergence hypothesis jointly, merge them to form the new Club 1, then run the log*t* test for the new Club 1 and the initial Club 3 jointly. If they do not fulfil the hypothesis, run the log*t* test for initial clubs 2 and 3, etc. The new club classifications would then be obtained by the above procedure. After this, we can also repeat the procedure on the newly obtained club classifications until no clubs can be merged, which ultimately gives classifications with the smallest number of convergence clubs.

## Data and variables

This paper analyses convergence in public health expenditure. One of the most important indicators of the level of healthcare financing is health spending per capita [38]. We used annual domestic general government health expenditure per capita, which represented public expenditure on health from domestic sources per capita, expressed in US dollars at purchasing power parity (PPP), and was provided by the *World Development Indicators* [39]. All the economies of SSA were studied, except for Zimbabwe, Somalia, and South Sudan, for which data are missing. Our sample was a balanced panel of 44 countries between 2000 and 2016, making a total of 748 observations.

Table 1 shows the sample. Countries are characterised by their organisation membership, their mean health expenditure over the study period, and the period of implementation of a national health policy. The national policies are focused on regional, continental or international targets. Indeed, there has been increased advocacy for UHC leading to the promotion of new health financing policies such as health insurance, user fee exemption and results-based financing. The promotion of UHC has led to the increased implementation of these three financing policies since 2010, particularly in SSA [40].

**Table 1:** Sample description

Country	Economic organisation membership	Health expenditure Mean (2000–2017)	National health policy implementation
Angola	COMESA	86,28	–
Benin	ECOWAS	16,40	2009–2018
Botswana	SADC	446,31	1995–2016
Burkina Faso	ECOWAS	21,02	2011–2020
Burundi	COMESA	14,73	2016–2025
Cabo Verde	ECOWAS	156,92	2008–2020
Cameroon	ECCAS	20,26	2011–2015
Cent. African Rep.	ECCAS	9,56	2004–2020
Chad	ECCAS	20,32	2007–2015
Comoros	COMESA	16,51	2005–2015
Congo. Dem. Rep.	COMESA	2,14	2011–2020
Congo. Rep.	ECCAS	43,89	2009
Cote d’Ivoire	ECOWAS	23,70	2011
Djibouti	COMESA	56,36	–
Equatorial Guinea	ECCAS	119,94	–
Eritrea	COMESA	12,38	2012–2016
Eswatini	COMESA	281,35	2007–2015
Ethiopia	COMESA	10,74	2012–2016
Gabon	ECCAS	221,54	2010–2020
Gambia. The	ECOWAS	24,25	2012–2020
Ghana	ECOWAS	67,98	2007
Guinea	ECOWAS	7,60	2015–2024
Guinea-Bissau	ECOWAS	30,51	2008–2017
Kenya	COMESA	38,82	2012–2020
Lesotho	SADC	95,83	2011–2020
Liberia	ECOWAS	8,93	2011–2021
Madagascar	COMESA	25,62	–
Malawi	COMESA	16,30	2011–2016
Mali	ECOWAS	18,66	2014–2023
Mauritius	COMESA	276,46	–
Mozambique	SADC	14,28	2014–2019
Namibia	COMESA	424,53	–
Niger	ECOWAS	13,82	2016–2035
Nigeria	ECOWAS	33,33	2004–2015
Rwanda	COMESA	24,07	2000–2020
Sao Tome and Princ.	ECCAS	61,75	2012–2022
Senegal	ECOWAS	34,90	2009–2018
Seychelles	COMESA	637,02	2015–2025
Sierra Leone	ECOWAS	16,68	–
South Africa	SADC	383,45	–
Sudan	COMESA	64,14	2016–2025
Tanzania	SADC	29,97	2007
Togo	ECOWAS	12,51	2011
Zambia	COMESA	45,63	2011–2020

We retrieved information on health policy implementation from the *Atlas of African Health Statistics* [41]. However, we did not find the implementation of any national health policy in eight countries of the sample. National health policies have been clearly defined and implemented as a whole over a period starting in 1995 and ending no later than 2020. In our study, five countries began implementing a national health policy towards the end of our research period (2000–2016).

## Results

Table 2 presents the full panel convergence test. The estimated value of *b* is −0.744 and the *t* statistic indicates that the parameter is significantly less than zero. We thus conclude for the divergence of the full group of 44 SSA countries. Panopoulou and Panteledis [26] found similar results.

**Table 2:** Full convergence test

log*t*	
*b*	*t*_*b*_
− 0.7442^a^	−45.5123

^a^Rejection of the null hypothesis of convergence at the 5% level

We then ran the algorithm of club clustering and club merging to examine whether there were any subgroups of countries that would converge. The results are presented in Table 3. Initially, the results of club clustering (Steps 1–4) indicate eight convergence clubs and one group of non-converging countries. The eight clubs of convergence consist of seven, three, two, four, seven, three, six and eight members, while the group of non-converging countries has four members. In this stage, the point estimate of *b* is positive for the converging clubs. For the non-converging club, it is negative and statistically different from zero, suggesting divergence. Our previous results of diverging countries are in line with the findings of Panopoulou and Panteledis [14]. The figures in Additional file 1 illustrate the relative transition paths for each of the eight converging clubs.

**Table 3:** Convergence club classification

Initial classification log*t*		Tests of club merging log*t*								Final classification log*t*	
Club 1 [7]	0.016	Club 1 + 2								Club 1 [12]	−0.004
	(0.321)	0.015									(−0.087)
Club 2 [3]	0.046	(0.320)	Club 2 + 3								
	(1.021)		0.039								
Club 3 [2]	1.971		(0.882)	Club 3 + 4							
	(11.532)			−0.039							
Club 4 [4]	0.199			(−0.990)	Club 4 + 5					Club 2 [14]	0.011
	(2.975)				0.212						(0.158)
Club 5 [7]	0.630				(2.351)	Club 5 + 6					
	(4.904)					0.182					
Club 6 [3]	0.161					(2.552)	Club 6 + 7				
	(2.184)						0.097				
Club 7 [6]	0.206						(1.159)	Club 7 + 8		Club 3 [14]	−0.021
	(1.757)							−0.021			(−0.192)
Club 8 [8]	0.219							(−0.192)	Club 8 + 9		
	(1.365)								−1.326^a^		
Group1 [4]	−1.733^a^								(− 174.920)	Group1 [4]	−1.733^a^
	(− 665.6)										(−665.59)

^a^ Rejection of the null hypothesis of convergence at the 5% level. The number of club members is reported in brackets. The ${\hat{t}}_b$ statistics are in parenthesis and under the associated coefficient $\hat{b}$ of log*t*

According to the algorithm, the club merging test is run on nested pairwise of the eight initial clubs and the group of non-converging countries. Testing for convergence among the initial clubs provided the results highlighted in the second column of Table 3. In this level, note that although the point estimate of *b* is negative for the merged Club 3 and 4 and the merged Club 7 and 8, the *t* statistic for each merged club indicates that both estimates are not statistically different from zero, suggesting convergence among the members of each merged club. The estimate of *b* is negative for the last merged club (initial Club 8 plus Group 9) and is significantly different from zero, suggesting that divergence still exists among members obtained when the initial non-converging club is merged with any converging club. Finally, in performing the club merging test, we determined the final club classification (third column of Table 3). The results indicate three final clubs: the first club (Club 1), consisting of initial clubs 1, 2 and 3, the second club (Club 2), consisting of initial clubs 4, 5 and 6 and the third club (Club 3) consists of the initial clubs 7 and 8. Therefore, the number of countries in the three clubs are 12, 14 and 14, respectively. The results also support the existence of the diverging group that was initially obtained (Group 1).

**Fig. 1:**
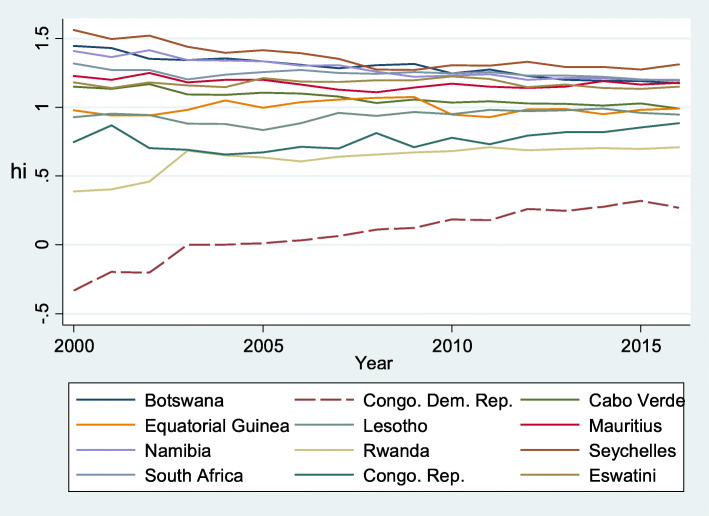
Convergence in Club 1

**Fig. 2:**
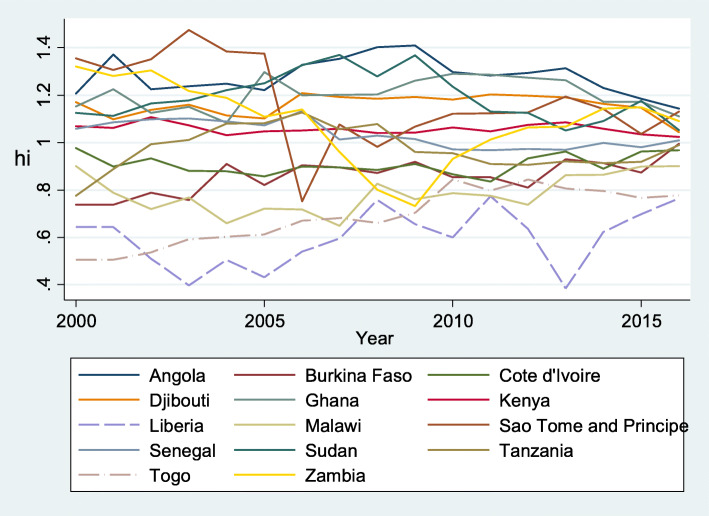
Relative convergence within Club 2

**Fig. 3:**
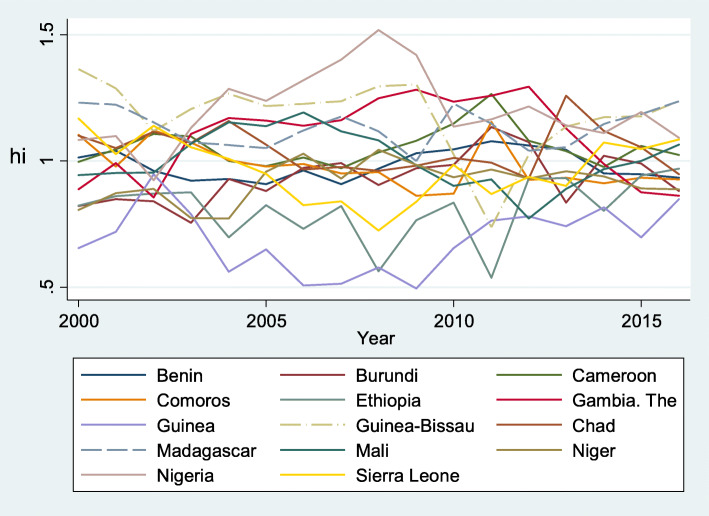
Relative convergence within Club 3

In summary, we obtained three convergence clubs (Club 1, Club 2 and Club 3) and one non-converging club (Group 1), as shown in Table 4. Figures 1, 2 and 3 represent the relative convergence paths of Club 1, Club 2 and Club 3 respectively. As a whole, Club 1 contains countries with higher per capita health expenditure than countries in Club 2 and Club 3. However, convergence among the members of Club 2 is proceeding faster than the convergence rates in the other clubs, as indicated by the higher estimate of *b* and illustrated by Fig. 2 compared with Figs. 1 and 3. When comparing the figures, we can observe that Club 2 seems to converge to the common steady state (Fig. 2), while the other clubs converge to a different steady state. However, the speeds of convergence among the members of Club 1 and Club 3 are slower, as indicated by the fact that the estimated *b* is negative and is not statistically different from zero. Club 1 converges faster than Club 3.

**Table 4:** Convergence clubs

Club 1 [12]	Club 2 [14]	Club 3 [14]	Group 1 [4]
Botswana	Angola	Benin	Eritrea
Congo. Dem. Rep.	Burkina Faso	Burundi	Gabon
Cabo Verde	Cote d’Ivoire	Cameroon	Cent. African Rep.
Equatorial Guinea	Djibouti	Comoros	Mozambique
Lesotho	Ghana	Ethiopia	
Mauritius	Kenya	Gambia. The	
Namibia	Liberia	Guinea	
Rwanda	Malawi	Guinea-Bissau	
Seychelles	Sao Tome and Princ.	Chad	
South Africa	Senegal	Madagascar	
Congo. Rep.	Sudan	Mali	
Eswatini	Tanzania	Niger	
	Togo	Nigeria	
	Zambia	Sierra Leone	

Number of club are in brackets

The group of diverging countries consists of Eritrea, Gabon, the Central African Republic and Mozambique. Gabon is a country with a high level of health expenditure that implemented a national health policy in the period 2010–2020. Eritrea, the Central African Republic and Mozambique are countries with low levels of health expenditure that implemented national health policies in the periods of 2012–2016, 2004–2020 and 2014–2019, respectively. Gabon and the Central African Republic are both members of ECCAS.

We then examined the level of health outcomes both within and across the three convergence clubs in the SSA countries. We linked the results of club convergence in health expenditure to infant mortality (ages five and below) in 2015 as an indicator of health performance (Table 5). The data were retrieved from the *Atlas of African Health Statistics* [41] and shows that Club 1, containing countries with high levels of health expenditure, had the lowest levels of infant mortality in 2015. Club 2, which contained countries converging faster and in the common steady state, had the second-lowest mortality rate according to the health performance indicator. For countries in this club, excluding Angola, the cross-country variation of infant mortality in this club seems to be lower.

**Table 5:** Health outcome of the convergence clubs

Clubs	Infant mortality (2015)	Clubs	Infant mortality (2015)
Club 1 [12]		**Club 3 [14]**	
Mauritius	14	Madagascar	50
Seychelles	14	Ethiopia	59
Cabo Verde	25	Gambia. The	69
South Africa	41	Comoros	74
Rwanda	42	Burundi	82
Botswana	44	Cameroon	88
Congo. Rep.	45	Guinea-Bissau	93
Namibia	45	Guinea	94
Eswatini	61	Niger	96
Lesotho	90	Benin	100
Equatorial Guinea	94	Nigeria	109
Congo. Dem. Rep.	98	Mali	115
Club 2 [14]		Sierra Leone	120
Sao Tome and Princ.	47	Chad	139
Senegal	47		
Kenya	49		
Tanzania	49		
Ghana	62		
Malawi	64		
Zambia	64		
Liberia	70		
Togo	78		
Burkina Faso	89		
Cote d’Ivoire	93		
Sudan	93		
Angola	157		
Djibouti	–		

Regarding this link between convergence in health expenditure over the period of 2000–2016 and the level of infant mortality in the year 2015, contrary to Panopoulou and Panteledis’ [14] findings, one could expect that, in the context of the SSA countries, convergence in health expenditure could lead to a convergence in health outcomes. The convergence clubs that are characterised with lower health expenditure may devote more federal resources to the health sector to improve the health of the population and rival the health of the populations of the countries in Club 1.

## Discussion

Our investigation of health expenditure convergence in SSA has provided some meaningful results, given the convergence paths of countries and their regional economic organisation memberships. The results could also highlight the different types of regional coordination regarding health expenditure policies in SSA.

The first set of results (Table 2) highlight that health expenditure in SSA displays a non-convergence picture. The absence of full convergence indicates idiosyncratic factors – which could be demographic, institutional, sociological or even geographical – for each country in the sample that maintains a different public health spending distribution. Such a result indicates that the SSA governments need to invest more money for greater involvement so that their health expenditure can lead to a convergence model. Indeed, Apergis [16]  highlights that fully functioning health systems can facilitate greater efficiency and production, since poorly functioning health systems are costly, unproductive and ultimately unsustainable. Therefore, the convergence of health spending in SSA could increase economies of scale in public health budget management and can increase the efficiency of healthcare systems across SSA.

The results also show the presence of three convergence clubs and one diverging group (Table 4), showing that SSA countries have taken a different approach in terms of their public health policies, with the governments contributing in different ways to their respective health sectors. The group of diverging countries consists of Eritrea, Gabon, the Central African Republic and Mozambique. Among these diverging countries, two countries (Gabon and the Central African Republic) are members of ECCAS, while Eritrea is a member of COMESA and Mozambique is a member of SADC). The findings of the diverging countries highlight that idiosyncratic factors (e.g., demographic, institutional, sociological and geographical factors) that maintain different public health spending distributions are both prominent and specific to these countries.

In Club 1, the converging club consisting of 12 countries, six are members of COMESA, and three (Botswana, Lesotho and South Africa) are members of SADC. Only one country (Cabo Verde) is a member of ECOWAS, and one country (Congo Republic) is a member of ECCAS. The governments of the SADC, ECOWAS and ECCAS countries that converge with the COMESA countries could incorporate very different health spending policies than the other members of their organisations.

Indeed, when the government is more involved in healthcare policies, their respective countries converge with SSA’s relatively high-income countries that make up COMESA (the Congo Democratic Republic, Botswana, Mauritius, Seychelles, Namibia, Eswatini).

In the second converging group (Club 2), which consists of 14 countries, six countries are members of COMESA and six are members of ECOWAS. One country (São Tomé and Principe) is a member of ECCAS, and one country (Tanzania) is part of SADC. The third convergence group also has 14 members. Among those, eight countries are members of ECOWAS, four countries (Burundi, Comoros, Ethiopia and Madagascar) are COMESA members, and two countries (Cameroon and Chad) are members of ECCAS.

In general, we found more evidence of convergence among countries within the same economic organisation in each of the three convergence clubs. COMESA countries seem to converge faster within a club, followed by ECOWAS countries. This result indicates that the SSA countries form three specific groups that are mostly characterised by individual, idiosyncratic factors that determine the course of their path regarding public health expenditure. The result also confirms that countries within the same regional economic organisation sometimes appear to have chosen dissimilar paths regarding their level of public health expenditure. Therefore, convergence was found to occur among different subgroups of economic organisation. However, according to Ham [42], we can determine that countries that form the same convergence club, or a particular group (such as a regional economic organisation) within a convergence club, have a similar period of national health policy implementation (see the last column of Table 1), namely the UHC programmes that have been widely implemented in several SSA countries and imply greater government involvement in healthcare financing. We can also assume that those countries are also characterised by similar tastes and technologies in their health sectors. Indeed, most countries in Club 1 have successfully implemented the UHC programme, while in the two other groups, the UHC has been implemented to lesser degrees, with a somewhat greater focus on mutual insurance.

The results also show that countries within Club 2 seem to converge faster than those in the other clubs. Similar to Apergis [16] findings, the differences across the clubs in terms of both speed of convergence and regional economic organisation could indicate different fiscal spending strategies that support human capital allocation across SSA’s regional economic organisation with the aim of achieving economic integration. Additionally, the results shown in Table 5 highlight potential inefficiencies in public investments on health. Indeed, the health outcomes (namely, the infant mortality rate) show several disparities both within and across the convergence clubs, leading us to question whether convergence in health expenditure leads to convergence in health outcomes in the SSA countries.

## Conclusion

This study contributes to the literature on SSA by examining convergence in domestic general health expenditure per capita across a panel of 44 countries, spanning the period 2000–2016. Our methodology has two important advantages [13]. Firstly, it allows for the possibility of transitional heterogeneity and remains robust to the stationarity properties of the series. Secondly, it is useful for identifying any subgroups among converging countries when there is a divergence in the panel of countries under scrutiny. Otherwise, the method also allows for individual countries to diverge.

Our results do not support the hypothesis that all SSA countries converge to a single equilibrium state regarding public health expenditure. Instead, we found evidence that three convergence clubs regarding health expenditure were present among SSA countries, consisting of 12, 14 and 14 members, respectively. Additionally, we found evidence for a group of four countries that diverged. Convergence does not solely occur among countries within the same regional economic organisation. The results highlight evidence of different regional economic organisations within the convergence clubs. Through our examination of the level of health performance within and across each convergence club, our results provide some eventuality of a correlation between club convergence in health expenditure and convergence in health performance (or health status) in SSA. Future research may be able to examine this assumption more rigorously. Our results can also contribute to the analysis and evaluation of the health policy choices of SSA countries.

## Supplementary Information

**Additional file 1.** The initial convergence clubs.

## Data Availability

Data used in this article are from two mains sources. First, the variable public domestic health expenditure are from the Word Band Indicators/World Bank National Accounts data available on http://datacatalog.worldbank.org. Second, ‘under-five’ (ages 5 years and below) infant mortality rate in the year 2015 as an indicator of health performance is retrieved from the *Atlas of african health statistics 2016: health situation analysis of the African Region*.

## Abbreviations

SSA: Sub-Saharan Africa
USA: United States of America
NCDs: Noncommunicable diseases
LM: Lagrange multiplier
OECD: Organization for Economic Co-operation and Development
PPP: Purchasing Power Parity
COMESA: Common Market for Eastern and Southern Africa
ECCAS: Economic Community of Central African States
ECOWAS: Economic Community of West African States
SADC: Southern African Development Community
UHC: Universal Health Coverage
Dem.: Democratic
Rep.: Republic
Cent.: Central
