# Supplementary figures and images for: Convergence in public health expenditure across the Sub-Saharan African countries: does club convergence matter?
Source: Health Econ Rev. 2021 Jun 15;11:21. doi: 10.1186/s13561-021-00316-0 (PMC8207633; doi:10.1186/s13561-021-00316-0)

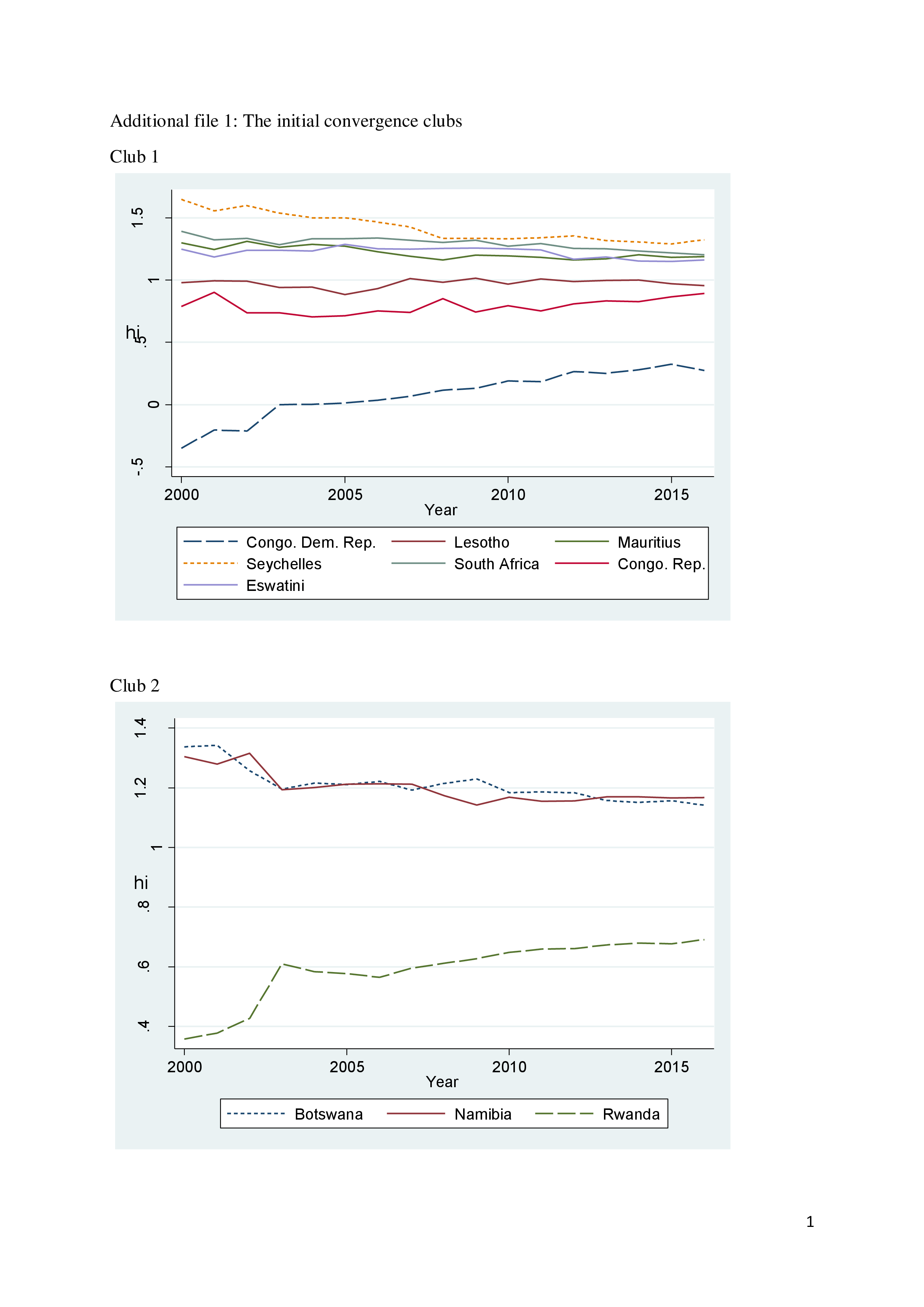

Supplement: Supplementary file 1 — Additional file 1. The initial convergence clubs. [file 13561_2021_316_MOESM1_ESM.tiff]
